# Acute esophageal necrosis in patients with diabetic ketoacidosis: a systematic review of clinical features and outcomes

**DOI:** 10.1007/s12328-026-02383-4

**Published:** 2026-07-06

**Authors:** Hideya Itagaki, Tomoyuki Endo

**Affiliations:** Division of Emergency and Disaster Medicine, Tohoku Medical and Pharmaceutical University Hospital, 1-12-1, Fukumuro, Miyaginoku, Sendai, Miyagi 983-8512 Japan

**Keywords:** Acute esophageal necrosis, Black esophagus, Diabetic ketoacidosis, Upper gastrointestinal bleeding, Systematic review

## Abstract

**Background:**

Acute esophageal necrosis (AEN) is a rare but serious disorder that occurs in critically ill patients. Although diabetic ketoacidosis (DKA) is a recognized precipitating factor, the clinical characteristics of DKA-associated AEN have not been well defined. This systematic review aimed to summarize the clinical presentation, endoscopic findings, management, and outcomes of AEN occurring in patients with DKA.

**Methods:**

A systematic literature search of PubMed and Web of Science was conducted from database inception to March 31, 2026, in accordance with PRISMA 2020. Eligible studies reported AEN confirmed by upper gastrointestinal endoscopy or postmortem examination occurring in association with DKA and provided sufficient case-level clinical data. Case reports and case series were included. Data were synthesized narratively.

**Results:**

Thirty-six studies involving 39 patients with DKA-associated AEN were included. The median age was 55.0 years (interquartile range [IQR], 49.0–65.0), and 29 patients (74.4%) were male. Overt upper gastrointestinal bleeding was reported in 28 of 32 evaluable cases (87.5%), although 10 patients (25.6%) developed bleeding only after admission. Shock or hypotension was present in 18 of 34 cases (52.9%). Endoscopy was performed in 34 cases (87.2%). AEN extent was evaluable in all 39 cases based on endoscopic or postmortem descriptions; 25 patients (64.1%) had mid/distal-predominant involvement, whereas 14 patients (35.9%) had involvement of the entire esophagus. Conservative treatment was the mainstay of management. In-hospital mortality was 13.5% (5/37).

**Conclusions:**

DKA-associated AEN frequently presents with overt upper gastrointestinal bleeding, although bleeding may also develop after admission. Awareness of this condition may help clinicians consider timely diagnostic evaluation and supportive management in high-risk patients with DKA.

**Supplementary Information:**

The online version contains supplementary material available at 10.1007/s12328-026-02383-4.

## Background

Acute esophageal necrosis (AEN) is a condition first described by Goldenberg et al. in 1990 and is also known as black esophagus or Gurvits syndrome [1, 2]. Although it is a rare disorder, with an estimated incidence of 0.28%, it can occur in critically ill patients and is associated with a high mortality rate [2, 3]. The pathophysiology of AEN is thought to involve a combination of esophageal ischemia, impairment of mucosal barrier function, and reflux-related mucosal injury [4, 5]. The etiology of AEN is usually multifactorial, and severe systemic conditions such as sepsis, hypotension/shock, alcohol intoxication, and hypothermia are recognized precipitating factors; diabetic ketoacidosis (DKA) is also considered one of the important causes [5, 6]. A recent systematic review suggested that patients with AEN accompanied by ketoacidosis may have higher survival rates than those without ketoacidosis [7]. However, in that analysis, different types of ketoacidosis, including diabetic, alcoholic, and starvation-related ketoacidosis, were evaluated together. As a result, the clinical characteristics, management patterns, warning symptoms, and outcomes of DKA-associated AEN have not yet been sufficiently clarified as a distinct emergency condition. The aim of this review was to characterize the clinical features and outcomes of DKA-associated AEN and to provide insights that may improve early recognition and management in emergency care.

## Methods

### Protocol and registration

This systematic review was conducted in accordance with the PRISMA 2020 (Preferred Reporting Items for Systematic Reviews and Meta-Analyses) guidelines. The review protocol was not pre-registered in PROSPERO or any other publicly accessible database. However, the study methodology, including the search strategy, inclusion criteria, and outcomes of interest, was defined a priori.

### Information sources and search strategy

A systematic literature search was conducted in PubMed and Web of Science from database inception to March 31, 2026.

PubMed was searched using AEN-related terms combined with DKA- and ketosis-related terms. The AEN-related terms included “acute esophageal necrosis,” “acute oesophageal necrosis,” “black esophagus,” “black oesophagus,” “Gurvits syndrome,” “necrotizing/necrotising esophagitis,” “necrotizing/necrotising oesophagitis,” and combinations of “esophagus” or “oesophagus” with “necrosis.” These were combined with terms including “Diabetic Ketoacidosis” [MeSH], “diabetic ketoacidosis,” “DKA,” “euglycemic/euglycaemic diabetic ketoacidosis,” “EDKA,” combinations of “diabet*” or “hyperglyc*” with “ketoacidosis,” “ketoacidotic,” “ketosis,” “ketotic,” “ketonemia/ketonaemia,” or “ketonuria,” and additional terms such as “diabetic ketosis,” “diabetic ketotic state,” “ketosis-prone diabetes,” “ketosis prone diabetes,” and “KPD.”

Web of Science was searched using AEN-related topic terms combined with DKA- and ketosis-related terms. The AEN-related terms included “acute esophageal necrosis,” “acute oesophageal necrosis,” “black esophagus,” “black oesophagus,” “Gurvits syndrome,” “necrotizing/necrotising esophagitis,” “necrotizing/necrotising oesophagitis,” and proximity combinations of “esophagus” or “oesophagus” with “necrosis.” These were combined with terms including “diabetic ketoacidosis,” “DKA,” “euglycemic/euglycaemic diabetic ketoacidosis,” “EDKA,” proximity combinations of “diabet*” or “hyperglyc*” with “ketoacidosis,” “ketoacidotic,” “ketosis,” “ketotic,” “ketonemia/ketonaemia,” or “ketonuria,” and additional terms such as “diabetic ketosis,” “diabetic ketotic state,” “ketosis-prone diabetes,” “ketosis prone diabetes,” and “KPD.”

The final search strategy included broader ketosis-related terms to improve sensitivity for identifying potentially relevant DKA-associated AEN cases. No additional eligible cases were identified within the predefined search period.

The titles and abstracts of all retrieved records were screened to identify potentially eligible studies. The full texts of potentially relevant articles were then assessed for eligibility. In addition, the reference lists of relevant articles were manually reviewed to identify any additional eligible studies.

### Eligibility criteria

We included studies published up to March 31, 2026, that met all of the following criteria: (1) acute esophageal necrosis (AEN) confirmed by upper gastrointestinal endoscopy or postmortem examination; (2) concurrent diabetic ketoacidosis (DKA), as defined by the diagnostic criteria reported in each study; and (3) availability of sufficient clinical data on presentation, diagnosis, treatment when applicable, and outcomes. Case reports and case series were eligible for inclusion. Review articles, editorials, conference abstracts, duplicate publications, and articles without peer review were excluded. Studies were also excluded if the association between AEN and DKA could not be clearly determined or if insufficient case-level information was available.

### Study selection

Two reviewers (HI and TE) independently screened the titles and abstracts of all identified records. Full-text articles of potentially relevant studies were retrieved and independently assessed for eligibility by the same reviewers according to predefined inclusion and exclusion criteria. Discrepancies were resolved through discussion until consensus was reached.

### Data extraction and data items

Two reviewers (HI and TE) independently extracted data from the included studies using a standardized, predefined data collection form. The extracted variables included publication characteristics (first author, publication year, country, study design, and sample size), patient demographics, diabetes-related characteristics, presenting symptoms, laboratory findings, endoscopic or postmortem findings, treatment details when applicable, complications, and clinical outcomes. The extent of AEN was categorized according to the anatomical distribution described in the original endoscopic or postmortem findings. Cases were classified as “mid/distal-predominant” when necrosis was described as involving the middle and/or distal esophagus without clear involvement of the entire esophagus, and as “entire esophagus” when diffuse involvement of the whole esophagus was described. In cases without ante-mortem endoscopy, AEN extent was classified only when the postmortem findings clearly described the anatomical distribution of esophageal necrosis. Cases with insufficient information on lesion extent were excluded from extent-specific analyses, and denominators were calculated using the number of evaluable cases for each variable.

### Definitions and diagnostic criteria

#### Definition of AEN

AEN was diagnosed based on characteristic endoscopic or postmortem findings of black-appearing esophageal mucosa, usually involving the distal esophagus or extending more proximally, with a clear demarcation at the gastroesophageal junction when described. In postmortem cases, macroscopic and/or histopathological findings consistent with acute esophageal necrosis were accepted. Alternative causes of esophageal black discoloration were excluded when necessary.

#### Definition of DKA

DKA was defined according to the diagnostic descriptions in each original report. In general, cases accompanied by hyperglycemia, metabolic acidosis, and ketonemia or ketonuria were considered DKA. When data were available, laboratory findings, including elevated blood glucose levels, decreased pH, decreased serum bicarbonate, increased anion gap, and positive ketone bodies, were confirmed.

#### Definition of DKA-related AEN

DKA-associated AEN was defined as AEN that developed during the course of DKA or was identified in close temporal and clinical association with DKA, including cases confirmed at autopsy in patients who died from DKA. The review included cases described by the original authors as DKA-associated AEN, as well as cases in which a clear association with DKA could be determined based on clinical course, laboratory findings, or postmortem biochemical evidence. Conversely, cases of ketoacidosis caused by conditions other than diabetes, such as alcoholic ketoacidosis or starvation ketoacidosis, were excluded.

Since this review is primarily based on case reports and case series, variations in diagnostic criteria and reporting details were anticipated across studies. Therefore, while respecting the diagnoses reported in the original studies, we verified the clinical information and laboratory findings, considering the objectives of this review.

For transparency, the basis for inclusion as DKA-associated AEN was classified as either “original authors’ description” or “reviewers’ judgment.” Cases were categorized as “original authors’ description” when the original report explicitly described AEN as occurring in association with DKA, ketoacidosis, or a hyperglycemic metabolic crisis. Cases were categorized as “reviewers’ judgment” only when the original authors did not explicitly state this association but the temporal and clinical relationship was considered clear by the reviewers.

#### Quality assessment

The methodological quality of the included case reports and case series was assessed using the CARE (CAse REport) guidelines. Each item on the 30-point CARE checklist was scored as 1 if adequately reported and 0 if not reported or unclear. The CARE scores were used to assess the completeness of case reporting and to guide the interpretation of findings, but not to exclude studies from the review. Two reviewers independently performed the assessment, and disagreements were resolved by consensus.

#### Synthesis of results

The extracted cases were summarized in tables according to clinical characteristics, treatment approaches, and outcomes. Because of the small number of cases and substantial heterogeneity among the included reports, the findings were synthesized narratively. No quantitative meta-analysis was performed.

#### Data analysis

Continuous variables were summarized as medians with interquartile ranges (IQRs), and categorical variables were summarized as frequencies and percentages. Percentages were calculated using the number of cases with available data for each variable. Because this review was based primarily on case reports and case series with heterogeneous and incompletely reported data, the analysis was conducted descriptively. No formal group comparisons or hypothesis testing were performed. Data tabulation and descriptive summaries were performed using Stata/MP 18.0 (StataCorp LLC, College Station, TX, USA).

## Results

### Study selection

**Fig. 1:**
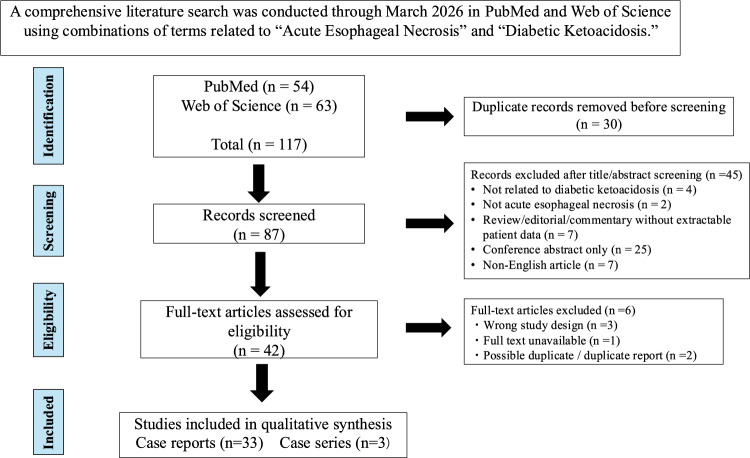
PRISMA flow diagram of study selection

A total of 117 records were identified through PubMed and Web of Science searches (Fig. 1).

A total of 117 records were identified through PubMed and Web of Science searches, including 54 from PubMed and 63 from Web of Science. After removing 30 duplicate records, 87 records remained for title and abstract screening. Following screening, 45 records were excluded because they were not related to diabetic ketoacidosis (n = 4), did not describe acute esophageal necrosis (n = 2), were review articles, editorials, or commentaries without extractable patient-level data (n = 7), were conference abstracts only (n = 25), or were non-English articles (n = 7). The full texts of 42 articles were then assessed for eligibility. Six articles were excluded after full-text review because of wrong study design (n = 3), unavailable full text (n = 1), or possible duplicate/duplicate reports (n = 2). Finally, 36 studies were included in the qualitative synthesis, consisting of 33 case reports and 3 case series. A total of 39 cases of DKA-associated AEN were identified from the included reports [8–43].

All results are presented as descriptive summaries, and denominators vary according to the availability of data for each variable. Regarding the basis for inclusion as DKA-associated AEN, most included cases were classified based on the original authors’ description. One case was included based on reviewers’ judgment because the original report did not explicitly describe the case as DKA-associated AEN, but the temporal and clinical relationship between DKA and AEN was considered clear based on the reported clinical course, laboratory findings, or postmortem findings. Cases with additional contributing factors, including alcohol intoxication, sepsis, hypothermia, hypertriglyceridemia, pancreatitis, or severe hyperglycemic metabolic crisis, were annotated in Supplementary Table 1.

### Quality assessment of included reports

The CARE scores of the included reports are summarized in Supplementary Table 2. The scores ranged from 16 to 28, with most reports rated as moderate to high quality. Lower scores were mainly observed in autopsy reports, clinical images, and letters because treatment details, patient perspective, and follow-up information were inherently limited.

### Baseline characteristics, comorbidities, and precipitating factors for DKA

Baseline characteristics, comorbidities, and precipitating factors for DKA are summarized in Table 1.

**Table 1:** Baseline characteristics, comorbidities, and precipitating factors for diabetic ketoacidosis in patients with acute esophageal necrosis

Parameters	Overall (n = 39)
*Clinical characteristics*	
Age, years [median (IQR)]	55.0 (49.0–65.0)
Sex, male, no. (%)	29 (74.4)
Known diabetes, no. (%)	33/36 (91.7)
Type 2 diabetes mellitus, no. (%)	17 (43.6)
Type 1 diabetes mellitus, no. (%)	8 (20.5)
Diabetes type not clearly reported, no. (%)	14 (35.9)
*Comorbidities and background conditions*	
Hypertension, no. (%)	15 (38.5)
Alcohol use present, no. (%)	8 (20.5)
Renal impairment, no. (%)	8 (20.5)
Upper gastrointestinal comorbidities or history, no. (%)	6 (15.4)
Cardiovascular disease, no. (%)	6 (15.4)
Dyslipidemia/hyperlipidemia, no. (%)	5 (12.8)
Liver disease, no. (%)	5 (12.8)
Pancreatic disease, no. (%)	4 (10.3)
Cerebrovascular disease, no. (%)	3 (7.7)
Respiratory disease, no. (%)	3 (7.7)
Malnutrition/undernutrition, no. (%)	3 (7.7)
Neuropathy, no. (%)	2 (5.1)
Gastroparesis, no. (%)	2 (5.1)
Congestive heart failure, no. (%)	2 (5.1)
Neuropsychiatric disease, no. (%)	2 (5.1)
Autoimmune disease, no. (%)	1 (2.6)
History of malignancy, no. (%)	1 (2.6)
Hypothermia, no. (%)	1 (2.6)
*Trigger for DKA*	
Insulin-related factors or poor glycemic control, no. (%)	13/39 (33.3)
Infection/sepsis, no. (%)	5/39 (12.8)
Drug- or diet-related factors, no. (%)	3/39 (7.7)
Dehydration/poor oral intake/nausea/vomiting, no. (%)	3/39 (7.7)
Alcohol-related factors, no. (%)	3/39 (7.7)
Not reported, no. (%)	12/39 (30.8)

Data are presented as no. (%) unless otherwise indicated. Percentages were calculated using the number of evaluable cases for each variable. Precipitating factors for DKA were categorized based on descriptions in the original case reports

DKA, diabetic ketoacidosis

The median age was 55.0 years (IQR, 49.0–65.0), and 29 patients (74.4%) were male. A history of diabetes was documented in 33 of 36 evaluable cases (91.7%). Regarding diabetes type, 17 cases (43.6%) were classified as type 2 diabetes mellitus, 8 cases (20.5%) as type 1 diabetes mellitus, and in 14 cases (35.9%), the diabetes type was not clearly specified or was reported only as insulin-dependent diabetes without further classification.

Among comorbidities and background conditions, hypertension was the most common, reported in 15 cases (38.5%), followed by alcohol use and renal impairment in 8 cases each (20.5%). Upper gastrointestinal comorbidities or history and cardiovascular disease were each reported in 6 cases (15.4%), whereas dyslipidemia/hyperlipidemia and liver disease were each reported in 5 cases (12.8%). Pancreatic disease was noted in 4 cases (10.3%), and cerebrovascular disease, respiratory disease, and malnutrition/undernutrition were each noted in 3 cases (7.7%).

Precipitating factors for DKA were diverse. Insulin-related factors or poor glycemic control were the most common, reported in 13 of 39 cases (33.3%), followed by infection or sepsis in 5 cases (12.8%). Drug- or diet-related factors, dehydration or poor oral intake/nausea/vomiting, and alcohol-related factors were each identified in 3 cases (7.7%). In 12 cases (30.8%), the precipitating factor was not clearly reported.

### Presenting features, laboratory findings, and endoscopic findings

The presenting features, laboratory findings, and endoscopic findings are shown in Table 2.

**Table 2:** Presenting features, laboratory findings, and endoscopic characteristics of diabetic ketoacidosis–associated acute esophageal necrosis

Parameters	Overall (n = 39)
*Presenting features*	
Overt upper gastrointestinal bleeding, no. (%)	28/32 (87.5)
Post-admission bleeding, no. (%)	10 (25.6)
Hematemesis, no. (%)	21/28 (75.0)
Melena, no. (%)	11/21 (52.4)
Chest pain, no. (%)	4/10 (40.0)
Epigastric pain, no. (%)	12/15 (80.0)
Odynophagia or dysphagia, no. (%)	7/12 (58.3)
Shock or hypotension, no. (%)	18/34 (52.9)
Sepsis or infection, no. (%)	9/27 (33.3)
*Laboratory findings at presentation*	
Initial glucose, mg/dL [median (IQR)]	665.0 (445.0–819.0)
Initial pH [median (IQR)]	7.19 (7.11–7.24)
Initial bicarbonate, mEq/L [median (IQR)]	9.0 (5.0–10.8)
Initial anion gap [median (IQR)]	27.5 (25.0–34.0)
Initial lactate, mmol/L [median (IQR)]	7.3 (2.4–10.1)
Initial creatinine, mg/dL [median (IQR)]	2.3 (1.6–3.1)
Initial BUN, mg/dL [median (IQR)]	76.0 (36.5–99.9)
*Endoscopic findings and complications*	
Endoscopy performed, no. (%)	34/39 (87.2)
AEN extent: entire esophagus, no. (%)	14/39 (35.9)
AEN extent: mid/distal-predominant, no. (%)	25/39 (64.1)
Duodenal involvement, no. (%)	6/28 (21.4)
Gastric involvement, no. (%)	11/28 (39.3)
Perforation, no. (%)	0 (0)
Pneumomediastinum, no. (%)	1/38 (2.6)
Stricture, no. (%)	5/26 (19.2)

Data are presented as median (interquartile range) for continuous variables and no. (%) for categorical variables, unless otherwise indicated. Percentages were calculated using the number of cases with available data for each variable. Overt upper gastrointestinal bleeding included hematemesis, coffee-ground emesis, melena, or other clearly described upper gastrointestinal bleeding manifestations. Post-admission bleeding was defined as overt gastrointestinal bleeding developing after hospital presentation or admission rather than as an initial presenting symptom. AEN extent was classified based on endoscopic or postmortem descriptions and was evaluable in all 39 cases

AEN, acute esophageal necrosis; DKA, diabetic ketoacidosis; BUN, blood urea nitrogen; IQR, interquartile range

Clinically overt upper gastrointestinal bleeding was present in 28 of 32 evaluable cases (87.5%). Hematemesis was reported in 21 of 28 cases (75.0%), and melena in 11 of 21 cases (52.4%). However, 10 cases (25.6%) developed gastrointestinal bleeding only after admission, indicating that upper gastrointestinal bleeding was not invariably the initial presentation. Other presenting symptoms included chest pain in 4 of 10 cases (40.0%), epigastric pain in 12 of 15 cases (80.0%), and odynophagia or dysphagia in 7 of 12 cases (58.3%).

Shock or hypotension was reported in 18 of 34 cases (52.9%), indicating frequent hemodynamic instability at presentation. Sepsis or infection was reported in 9 of 27 cases (33.3%), suggesting that systemic inflammatory or infectious conditions were also common among the reported cases.

Laboratory findings at presentation were consistent with severe DKA. The median initial glucose level was 665.0 mg/dL (IQR, 445.0–819.0), the median pH was 7.19 (IQR, 7.11–7.24), the median bicarbonate level was 9.0 mEq/L (IQR, 5.0–10.8), and the median anion gap was 27.5 (IQR, 25.0–34.0). In addition, the median lactate level was 7.3 mmol/L (IQR, 2.4–10.1), the median creatinine level was 2.3 mg/dL (IQR, 1.6–3.1), and the median blood urea nitrogen level was 76.0 mg/dL (IQR, 36.5–99.9).

Upper gastrointestinal endoscopy was performed in 34 of 39 cases (87.2%). AEN extent was evaluable in all 39 cases. In the five cases without ante-mortem endoscopy, the extent was classified based on postmortem descriptions that clearly described the anatomical distribution of esophageal necrosis. Based on the original endoscopic or postmortem descriptions, 25 of 39 cases (64.1%) showed mid/distal-predominant involvement, whereas 14 of 39 cases (35.9%) involved the entire esophagus.　Associated lesions were also reported in some cases. Duodenal involvement was present in 6 of 28 evaluable cases (21.4%), and gastric involvement in 11 of 28 cases (39.3%). No perforation was reported; pneumomediastinum occurred in 1 of 38 evaluable cases (2.6%), and stricture developed in 5 of 26 cases (19.2%).

### Management and outcomes

Management and outcomes are shown in Table 3. Management was primarily conservative, with proton pump inhibitors used in 33 of 37 cases (89.2%), total parenteral nutrition or bowel rest in 20 of 27 cases (74.1%), antibiotics in 20 of 37 cases (54.1%), and sucralfate in 10 of 23 cases (43.5%).

**Table 3:** Management strategies and clinical outcomes of diabetic ketoacidosis–associated acute esophageal necrosis

Parameters	Overall (n = 39)
ICU admission, no. (%)	14/20 (70.0)
Mechanical ventilation, no. (%)	3/14 (21.4)
Proton pump inhibitor use, no. (%)	33/37 (89.2)
Sucralfate use, no. (%)	10/23 (43.5)
Antibiotic use, no. (%)	20/37 (54.1)
TPN or bowel rest, no. (%)	20/27 (74.1)
Vasopressor use, no. (%)	4/23 (17.4)
Surgery or intervention, no. (%)	5/35 (14.3)
Length of hospital stay, days [median (IQR)]	11.0 (9.0–17.5)
In-hospital mortality, no. (%)	5/37 (13.5)

Data are presented as no. (%) unless otherwise indicated. Percentages were calculated using the number of cases with available data for each variable. In-hospital mortality was defined as death during the index hospitalization when reported

ICU, intensive care unit; IQR, interquartile range; TPN, total parenteral nutrition

ICU admission was reported in 14 of 20 cases (70.0%), mechanical ventilation in 3 of 14 cases (21.4%), and vasopressor use in 4 of 23 cases (17.4%). Surgery or other intervention was performed in 5 of 35 cases (14.3%). The length of hospital stay was available or could be reasonably converted to days in 14 cases, with a median of 11.0 days (IQR, 9.0–17.5). In-hospital mortality was reported in 5 of 37 cases (13.5%).

## Discussion

In this systematic review, we collected and analyzed published case reports of DKA-associated AEN to clarify its clinical characteristics, management patterns, and outcomes. The main findings were as follows. First, overt upper gastrointestinal bleeding was frequently observed in DKA-associated AEN, although some patients had no obvious bleeding at presentation and developed bleeding only after admission. Second, the anatomical distribution of AEN, based on endoscopic or postmortem descriptions, most commonly showed a mid-to-distal esophageal pattern, whereas in more than one-third of cases, it involved the entire esophagus. These findings should be interpreted as descriptive summaries of published cases rather than epidemiological estimates of DKA-associated AEN.

One of the most notable findings of this review was the high frequency of overt upper gastrointestinal bleeding in DKA-associated AEN. Upper gastrointestinal bleeding has also been reported as the most common symptom in the large systematic review of AEN cases by Kupferman et al., suggesting that it is a central clinical manifestation of this condition [7]. In that review, the frequency of gastrointestinal bleeding was 66.5%, whereas it was higher in our review at 87.5%, suggesting that bleeding symptoms may be more prominent in DKA-associated AEN. Furthermore, although the timing of bleeding onset was not examined in the Kupferman et al. review, our review showed that bleeding symptoms became apparent only after admission in 25.6% of cases. Therefore, even in DKA patients without overt gastrointestinal bleeding at presentation, AEN should still be considered.

Another notable finding of this review was the anatomical pattern of disease involvement. AEN is typically characterized by necrosis of the distal esophageal mucosa with a clear demarcation at the gastroesophageal junction [1]. One proposed reason why the distal esophagus is preferentially affected is that, unlike the more richly vascularized proximal esophagus, the distal esophagus is more commonly supplied by branches of the left gastric artery or left inferior phrenic artery and is therefore more vulnerable to ischemic injury [4]. In our review, mid-to-distal predominant involvement was the most common pattern, which was broadly consistent with this classic distribution. However, in more than one-third of cases, the lesion involved the entire esophagus, suggesting that DKA-associated AEN does not always present as the typical distal-limited form. Such extensive involvement may reflect the contribution of circulatory failure, vascular risk factors, and the pathophysiological effects of DKA itself.

AEN is a multifactorial condition and can occur outside the setting of ketoacidosis. In the previous systematic review by Kupferman et al., AEN cases not associated with ketoacidosis were clinically heterogeneous and were reported in association with various precipitating factors and background conditions, including shock, sepsis, hypovolemia, vascular comorbidities, chronic kidney disease, malignancy, alcohol use disorder, recent surgery or invasive procedures, and mucosal injury related to gastric contents, vomiting, medications, or other insults [7]. Therefore, DKA should be regarded as one of several potential precipitating clinical contexts for AEN rather than an exclusive cause.

In our review, shock or hypotension was present in 52.9% of evaluable cases, and vasopressor use was reported in 17.4%. In addition, background factors suggestive of vascular vulnerability, such as hypertension, renal impairment, cardiovascular disease, and dyslipidemia, were not uncommon. DKA itself is also recognized as a potential trigger for AEN. DKA may reduce circulating plasma volume through osmotic diuresis, and it is also known to promote gastric stasis and gastroesophageal reflux, thereby aggravating subsequent esophageal mucosal injury [4]. Taken together, these findings suggest that in DKA-associated AEN, more extensive esophageal involvement may result from the combined effects of systemic hypoperfusion, vascular vulnerability, and local mucosal injury related to reflux and vomiting.

From this pathophysiological perspective, an important clinical question is which patients with DKA should raise suspicion for AEN. In this regard, Iwamoto et al. reported in a comparative study of patients with DKA that those with concomitant AEN were more likely to have a background of heavy alcohol consumption, prolonged fasting, and sodium-glucose cotransporter 2 inhibitor use, and were significantly more likely to present with vomiting [6]. In our review, alcohol use, poor oral intake, nausea, and vomiting were also relatively frequent, and these findings were broadly consistent with the clinical profile of DKA-associated AEN described by Iwamoto et al. Therefore, in patients with DKA, the possibility of AEN should be actively considered when gastrointestinal symptoms are prominent or when there are background factors such as dehydration, fasting, or alcohol use.

From the standpoint of management and outcomes, treatment of DKA-associated AEN was primarily conservative. In our review, proton pump inhibitors, total parenteral nutrition or bowel rest, antibiotics, and sucralfate were frequently used, indicating that supportive care was the mainstay of treatment, as in previous reports. On the other hand, ICU admission was required in 70.0% of evaluable cases, suggesting that DKA-associated AEN often occurs as part of a severe systemic condition requiring intensive management. Surgical or other invasive interventions were performed in only a minority of cases; however, in-hospital mortality was still observed in 13.5%, indicating that this condition does not necessarily follow a benign course. Although the predominance of conservative management was consistent with the Kupferman et al. review, the observed mortality in our review was lower than that reported in previous broader AEN reviews. However, direct comparison is limited because of differences in study populations, case selection, reporting patterns, and the descriptive nature of the available data.

This review has several limitations. First, because this review was based almost entirely on published case reports and case series, the reported proportions should not be interpreted as epidemiological frequencies or incidence estimates of DKA-associated AEN. Rather, these values reflect the characteristics of cases reported in the literature and are therefore susceptible to publication bias, reporting bias, and selective reporting of clinically severe or unusual presentations. Second, there was substantial variability across reports in the details of diagnostic criteria, reported variables, and treatment descriptions, and missing data were common for several variables. The CARE assessment also indicated variability in reporting completeness across studies. Therefore, variables that depend heavily on detailed case reporting, such as treatment details, follow-up findings, and complications, should be interpreted with particular caution. Third, this review was descriptive and did not establish causal relationships among risk or prognostic factors for DKA-associated AEN. Accordingly, our findings should be interpreted as an effort to characterize the clinical profile of DKA-associated AEN and to generate hypotheses for future study.

## Conclusions

DKA-associated AEN frequently presents with overt upper gastrointestinal bleeding, although some patients do not have obvious bleeding at the time of presentation and may develop bleeding after admission. Endoscopically or postmortem, the disease most commonly shows mid/distal-predominant involvement, but extensive involvement of the entire esophagus is also observed in more than one-third of cases. In patients with DKA, awareness of this condition may be warranted, particularly when prominent gastrointestinal symptoms or background factors such as dehydration, prolonged fasting, or alcohol use are present.

## Supplementary Information

Below is the link to the electronic supplementary material.

Supplementary Material 1.

Supplementary Material 2.

## Abbreviations

AEN: Acute esophageal necrosis
CT: Computed tomography
DKA: Diabetic ketoacidosis
GI: Gastrointestinal
ICU: Intensive care unit
IQR: Interquartile range
PPI: Proton pump inhibitor
SGLT2: Sodium-glucose cotransporter 2
TPN: Total parenteral nutrition

## Declarations

### Generative AI and AI-assisted technologies

During the preparation of this work, the authors used ChatGPT (OpenAI) to assist with language editing and improve clarity. After using this tool, the authors reviewed and edited the manuscript and take full responsibility for its content.
